# Cold Plasma‐Mediated Seed Priming: Plant Stress Responses and Potential Ecological Implications

**DOI:** 10.1002/pei3.70228

**Published:** 2026-10-30

**Authors:** Javad Karimi, Saeideh Arbabi

**Affiliations:** ^1^ Department of Biology, College of Science Shiraz University Shiraz Iran

**Keywords:** abiotic stress, biotic stress, cold plasma, defense mechanisms, reactive species, seed germination, sustainable agriculture

## Abstract

Cold plasma (CP) is being investigated as a non‐thermal seed treatment because studies have reported effects on germination, early seedling growth, plant responses to stress, and seed‐borne microorganisms. These effects have been linked to the generation of reactive oxygen and nitrogen species (ROS and RNS), changes in seed‐surface characteristics, and alterations in physiological and biochemical processes within the plant. However, the response to treatment is not always consistent. It depends on factors such as plant species, plasma source, exposure time, and treatment intensity, while excessive exposure may adversely affect seed performance. Research to date suggests that CP may help improve crop establishment and modify plant responses to biotic and abiotic stresses. However, whether these effects lead to broader ecological benefits remains unclear. Relatively little is known about how CP treatment affects plant–microbe interactions, soil microbial communities, plant community structure, or ecosystem functioning under field conditions. It is also uncertain whether treatment‐induced responses persist across generations or involve stress memory and epigenetic regulation. These possibilities require direct experimental evidence rather than inference from short‐term physiological responses. This review examines current findings on the effects of CP on seed germination, pathogen suppression, and plant responses to environmental stress. It considers the mechanisms proposed to explain these effects, the variation observed across experimental systems, and the extent to which existing evidence supports the conclusions drawn. Potential ecological implications and unresolved evolutionary questions are also discussed. An important limitation of the current literature is its reliance on laboratory and controlled‐environment experiments, alongside a shortage of long‐term field studies and multigenerational investigations. As a result, the wider ecological consequences of CP seed treatment cannot yet be established with confidence. Addressing this gap will require more consistent treatment protocols, validation under field conditions, and studies that examine whether physiological and molecular responses are accompanied by measurable changes in plant performance, biological interactions, and ecosystem processes. Such evidence will be essential for determining both the practical value of CP seed treatment and its longer‐term environmental implications.

## 1 Introduction

Seed germination is a fundamental biological process that initiates the plant life cycle and plays a critical role not only in agricultural productivity but also in ecological processes such as species establishment, population dynamics, and ecosystem resilience. This process is essential for the conservation and restoration of plant species. In natural and managed ecosystems, germination success directly influences plant community structure, competitive interactions among species, and biodiversity patterns. Variations in germination timing and success can determine species dominance, niche occupation, and long‐term vegetation dynamics. Various factors, such as seed quality, environmental conditions (temperature, humidity, light), and sowing depth, can influence germination rates. Successful germination leads not only to proper plant establishment but also shapes ecological interactions and contributes to ecosystem functioning, including nutrient cycling and trophic relationships (Whitehouse et al. 2020).

Throughout their life cycle, plants are exposed to a wide range of biotic and abiotic stresses that influence not only their growth and yield but also their ecological fitness, survival, and distribution. These stress factors can alter interspecific competition, plant–microbe interactions, and community composition, ultimately affecting ecosystem stability and resilience. Stress tolerance refers to a plant's ability to survive and grow under adverse environmental conditions, mediated through mechanisms such as gene expression regulation, production of secondary metabolites, and activation of defense pathways. Enhancing stress tolerance in plants is therefore important not only for improving crop performance but also for maintaining ecological balance and adaptive capacity under changing environmental conditions (Mishra et al. 2023).

Plasma, known as the fourth state of matter, is an ionized gas composed of excited atoms, ions, electrons, and reactive species with a wide energy spectrum (Randeniya and de Groot 2015). It is generally categorized into thermal and non‐thermal (cold) plasma, which differ primarily in temperature and associated thermal effects (Mahendran et al. 2017). Among these, cold plasma has attracted considerable attention due to its low‐temperature nature, allowing biological applications without causing thermal damage to plant tissues or seeds. Cold plasma has emerged as a promising non‐thermal technology in plant science, demonstrating significant potential to enhance seed germination, plant growth, and resistance to environmental stresses (Barjasteh et al. 2021). Cold plasma technology can be applied directly or indirectly, depending on whether seeds or plants are exposed directly or via plasma‐activated media, and both approaches can influence plant physiological responses (Starič et al. 2020).

Cold plasma treatment has been reported to influence seed germination, early plant growth, and several physiological and biochemical processes associated with stress responses. The magnitude and direction of these effects vary with plant species, seed characteristics, plasma source, and treatment conditions. Although changes in germination, antioxidant activity, and pathogen load have been documented in experimental studies, their broader ecological significance remains less clear. In particular, improved seedling performance does not necessarily translate into greater competitive ability, changes in plant community composition, or improved ecosystem functioning. These outcomes depend on interactions among plants, microorganisms, and environmental conditions that cannot be fully assessed in most laboratory experiments. Accordingly, the ecological and evolutionary implications of cold plasma treatment should be regarded as questions for further investigation rather than established consequences of the technology (Priatama et al. 2022).

Beyond agricultural applications, cold plasma has been widely used in fields such as medicine for tissue disinfection, the food industry for reducing microbial load, air and water purification, and tissue engineering, highlighting its broad potential as a versatile and environmentally friendly technology (Mahendran et al. 2017). Given these characteristics, this review examines the effects of cold plasma on seed germination, pathogen control, and the enhancement of plant tolerance to abiotic stresses, with a focus on the underlying cellular and biochemical mechanisms. It also highlights key knowledge gaps and outlines future research directions, particularly in relation to ecological implications and large‐scale agricultural applications. Figure 1 presents a conceptual overview of how cold plasma treatment integrates into agricultural systems to improve seed quality, stress resilience, and broader ecosystem functioning.

**FIGURE 1:**
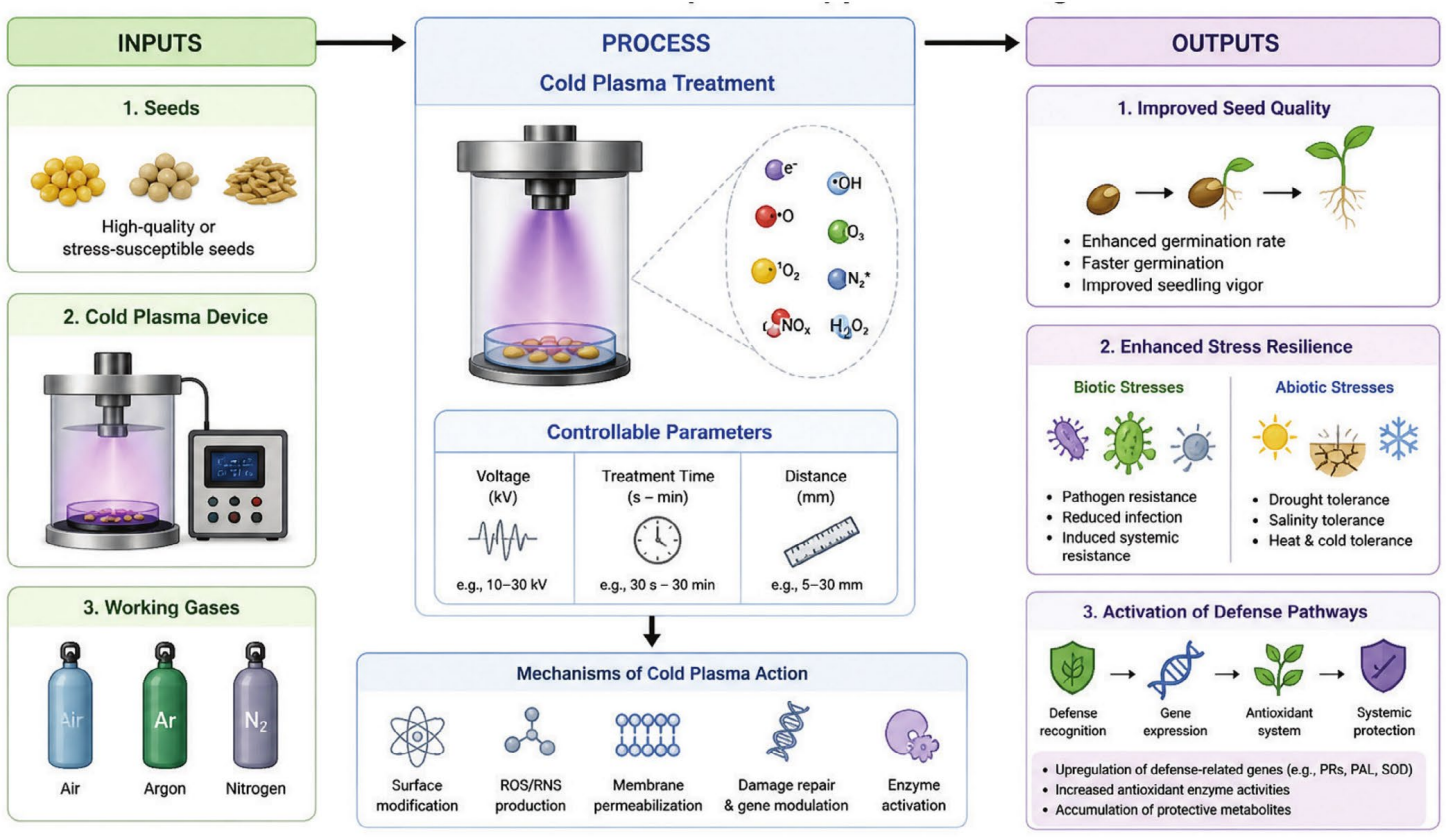
Overview of how cold plasma treatment integrates into agricultural systems to address both seed quality and stress resilience.

### 1.1 Evidence Strength and Ecological Interpretation

The effects of cold plasma treatment need to be considered in the context of the biological responses that have been examined. Previous studies have mainly focused on seed germination, seedling growth, changes in seed‐surface properties, and physiological and biochemical responses associated with stress tolerance. However, the outcomes are not always consistent and depend on several factors, particularly plant species and plasma treatment conditions. Improvements in germination or early seedling growth alone do not necessarily indicate corresponding benefits at the ecological level. Changes in plant community composition, interactions among species, soil microbial communities, and overall ecosystem functioning must be investigated separately under relevant environmental conditions. Similarly, stress memory and epigenetic regulation may help explain whether plasma‐induced responses persist over time, but the possibility of transgenerational inheritance requires direct evidence from successive generations and appropriate controls. In this review, we therefore distinguish between physiological effects demonstrated experimentally and the broader ecological and evolutionary implications that remain to be investigated.

To avoid overinterpretation, we distinguish between experimentally established findings and hypothetical or theoretical projections. Established findings include the generation of ROS/RNS and UV/electric fields by cold plasma, modification of seed‐surface chemistry and wettability, acceleration of germination and early growth under defined conditions, reduction of seed‐borne fungal and bacterial loads, and modulation of antioxidant enzymes and defense‐related genes in controlled experiments. In contrast, whether cold plasma treatment alters plant–microbe interactions, soil microbial communities, plant community structure, biodiversity, nutrient cycling, or ecosystem resilience remains hypothetical. Likewise, potential ecological trade‐offs, such as short‐term pathogen suppression versus effects on beneficial seed or rhizosphere microorganisms, and evolutionary consequences, including stress memory, epigenetic inheritance, transgenerational effects, adaptive plasticity, and natural selection, are plausible but not yet demonstrated. These are presented as hypotheses requiring direct experimental validation rather than established outcomes.

The available evidence can be considered at three interconnected levels. At the first level, cold plasma produces changes in seed‐surface properties and may alter germination, seedling growth, and physiological responses to stress. These responses are the principal outcomes examined in the current literature. At the second level, the resulting changes in plant establishment and performance could influence interactions with pathogens, beneficial microorganisms, neighboring plants, and environmental resources. Such effects are biologically plausible, but their direction and magnitude cannot be inferred from measurements of individual plants alone. At the third level, persistent changes in plant performance and biotic interactions could potentially affect population dynamics, plant community composition, or ecosystem processes. Evidence connecting cold plasma treatment to these broader outcomes remains limited. This framework highlights the need to move beyond measurements of germination and seedling physiology and to test explicitly whether the observed responses persist under field conditions and translate into measurable ecological effects.

To place the physiological effects of cold plasma treatment in a broader biological context, it is useful to consider how responses observed at the seed and individual‐plant levels might relate to processes operating at the population, community, and ecosystem levels. Changes in germination, seedling growth, and stress responses could potentially influence interactions between plants and microorganisms, as well as competition among neighboring plants. However, such effects cannot be assumed from physiological measurements alone, and their wider ecological significance remains unclear. Figure 2 presents a conceptual framework linking the responses directly associated with cold plasma treatment to possible ecological and evolutionary implications, while highlighting the need for further experimental evidence to establish these connections.

**FIGURE 2:**
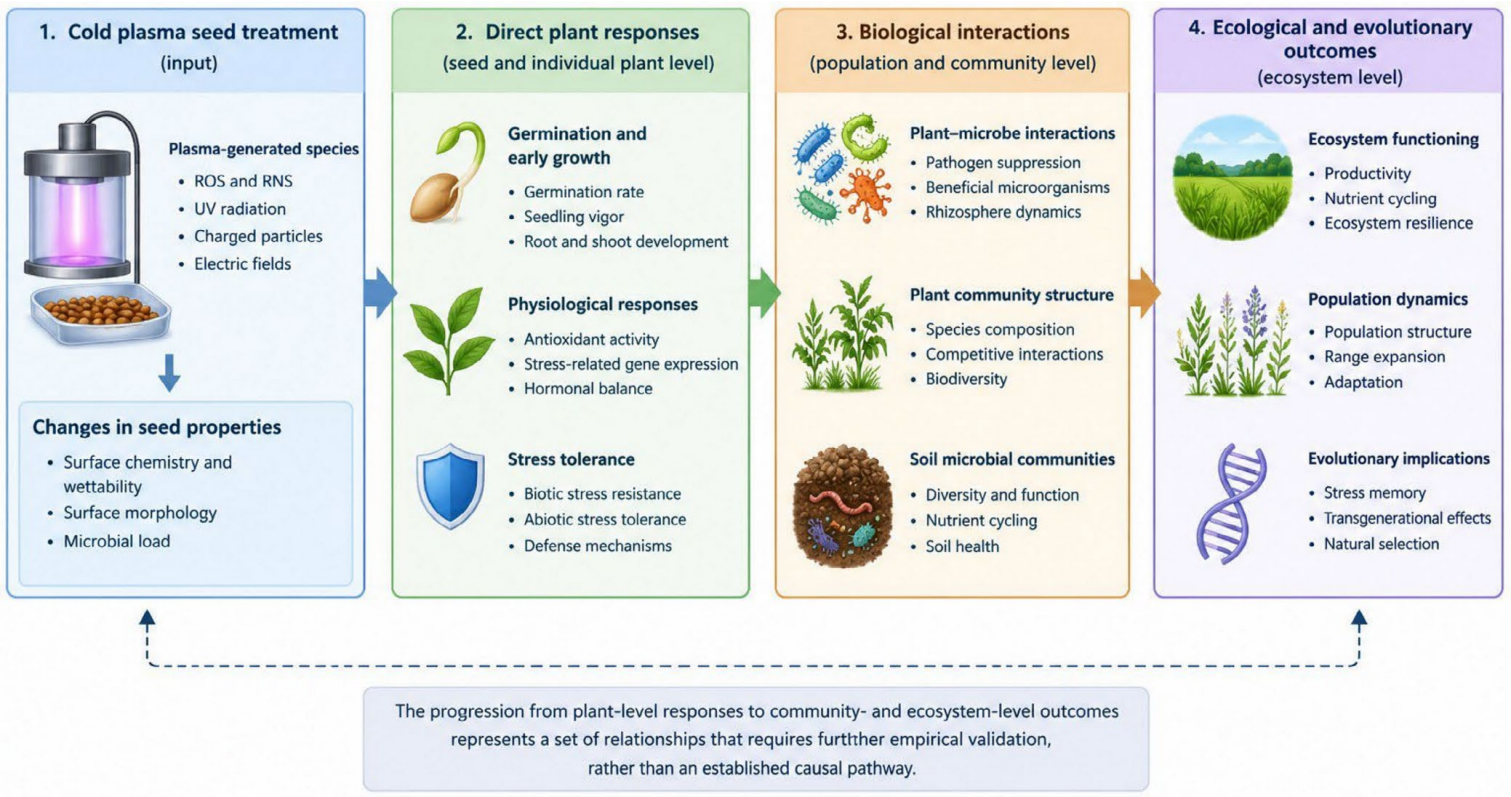
Conceptual framework linking direct physiological responses to cold plasma seed treatment with potential ecological consequences. The progression from plant‐level responses to community‐ and ecosystem‐level outcomes represents a set of relationships that requires further empirical validation, rather than an established causal pathway.

## 2 Effects of Cold Plasma on Improving Seed Germination

Seed germination is essential for the survival and persistence of plant species and plays a key role in shaping plant establishment and community dynamics. Germination is a complex process that begins with water uptake (imbibition) and the activation of metabolic and physiological pathways, ultimately leading to the breaking of seed dormancy and the initiation of seedling growth (Strejckova et al. 2016). Rapid and uniform germination is a critical factor for optimal crop establishment and early plant performance. Numerous studies have shown that cold plasma positively influences seed germination and subsequent plant growth. Upon contact with the seed surface, plasma induces structural and chemical modifications in the seed coat, enhancing permeability and reducing the time required for germination, thereby promoting faster and more uniform seedling development (Bafoil et al. 2018).

Seeds are often exposed to environments containing particles, pollutants, and microorganisms that can adversely affect germination. Cold plasma treatment can effectively inactivate microbial contaminants on seed surfaces, thereby reducing infection risks and enhancing germination performance (Guo et al. 2022). Plasma treatment enhances water uptake, modifies seed coat structure, increases permeability, breaks dormancy, and elevates the levels of sugars and soluble proteins, ultimately stimulating germination and early plant growth and contributing to improved crop development. The effectiveness of these responses depends on treatment parameters such as plasma intensity and exposure time, which vary across different plant species and therefore require careful optimization (Priatama et al. 2022). The mechanisms underlying the effects of cold plasma on seeds are summarized in Figure 3.

**FIGURE 3:**
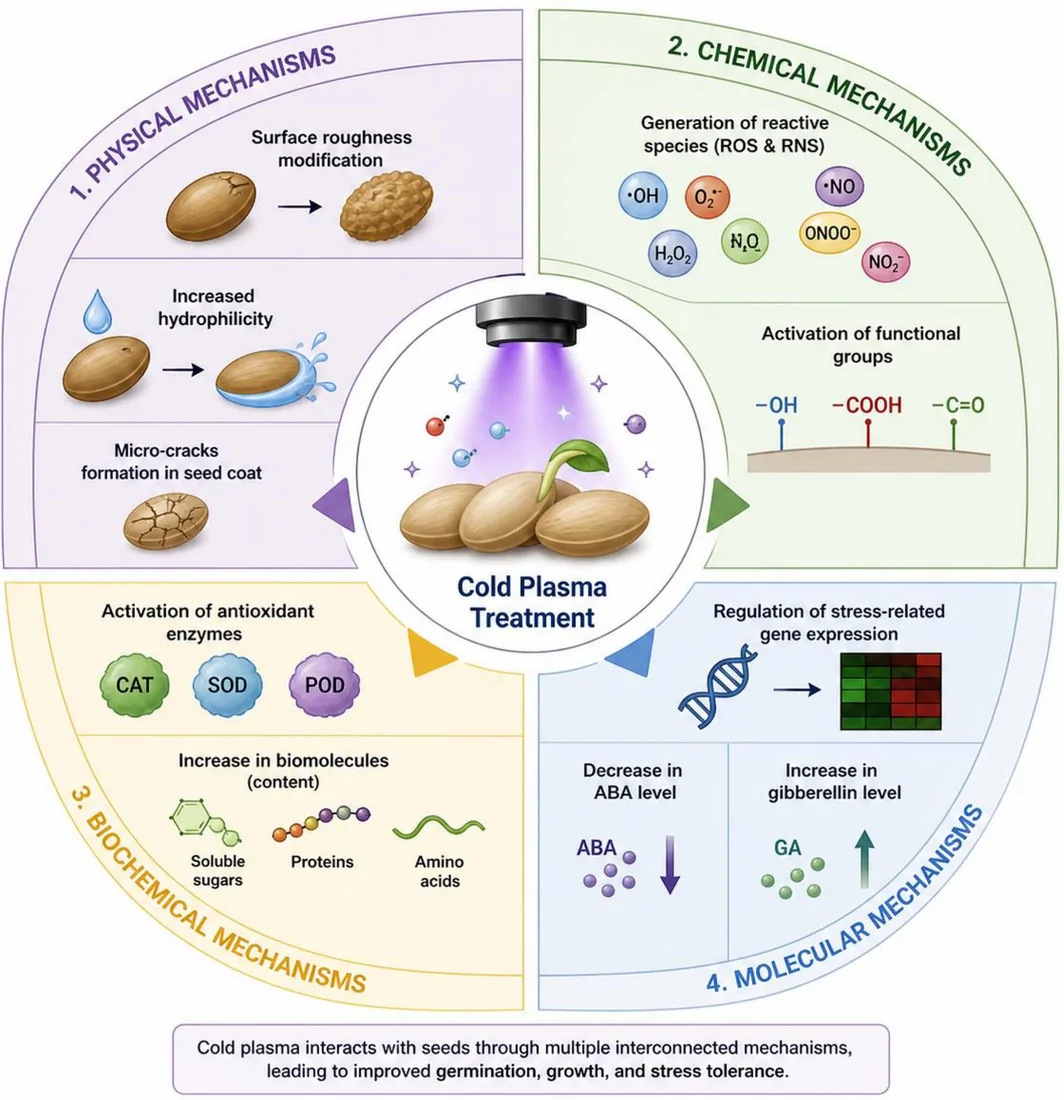
A summary of the mechanisms by which cold plasma affects seeds.

## 3 Cold Plasma Effects on Seed Germination

Seeds may enter a dormancy state under unfavorable environmental conditions such as drought, low temperature, or limited light availability. This adaptive mechanism prevents germination until conditions become suitable, thereby enhancing species survival, particularly in environments exposed to biotic and abiotic stresses. Dormancy is naturally regulated by environmental cues, including rainfall, temperature fluctuations, light exposure, and biotic interactions. For instance, repeated rainfall can soften the seed coat and promote water uptake, initiating germination, while temperature and light signals can trigger physiological pathways that lead to dormancy release (Mokrani and Tarchoun 2022).

### 3.1 Mechanisms of Cold Plasma Effects on Seeds

Cold plasma influences seed germination through a combination of physical, chemical, and biochemical mechanisms. One of the primary physical effects is the modification of the seed coat structure, including increased surface roughness and microstructural changes, which enhance water absorption and improve gas exchange, thereby facilitating more efficient germination processes (Randeniya and de Groot 2015). Plasma treatment can also reduce or eliminate inhibitory compounds present in the seed coat, thereby facilitating dormancy release. From a chemical perspective, reactive oxygen species (ROS) and reactive nitrogen species (RNS) generated by cold plasma induce oxidation of the seed surface and activate functional groups such as hydroxyl (–OH) and carboxyl (–COOH), which enhance water and nutrient uptake and support early metabolic activation (Ling et al. 2014).

These reactive species may also act as signals that regulate enzymes involved in germination and early seedling development. The resulting physiological response depends on treatment intensity, as moderate exposure may stimulate metabolic activity, whereas excessive exposure can cause oxidative damage (Pauzaite et al. 2018; Sivachandiran and Khacef 2017).

Several studies have demonstrated these effects. For instance, Sera et al. (2009) reported that cold plasma treatment modified germination characteristics in *Chenopodium album* , effectively eliminating irregular dormancy without negatively affecting the plant's biological traits (Sera et al. 2009). August et al. (2023) used atmospheric cold plasma to reduce dormancy in *Arabidopsis thaliana* (August et al. 2023), while Cui et al. (2019) observed increased superoxide anion and nitric oxide levels in plasma‐treated seeds, which were associated with dormancy release and germination. However, excessive treatment can impair germination, highlighting the importance of optimizing exposure conditions (Cui et al. 2019). Ozone generated by cold plasma can produce ethylene and remove abscisic acid (dormancy hormone), promoting germination (Emberson et al. 2018).

Seed coat changes are critical for germination, influencing nutrient and water transport, protection against environmental hazards, and dormancy regulation (Radchuk and Borisjuk 2014). Cold plasma enhances seed coat permeability, improving water and mineral absorption, thereby stimulating germination and growth (Waskow et al. 2021). Plasma also increases hydrophilicity and reduces contact angles, accelerating germination and enzyme activities such as amylase and protease, while reducing pathogen load (Pańka et al. 2022).

For example, Sadhu et al. (2017) reported that cold plasma significantly increased germination rate, root length, and seedling vigor in mung beans. Treated wheat seeds absorbed more water than controls, with higher plasma intensity and exposure time enhancing water uptake (Sadhu et al. 2017). Table 1 shows the effects of cold plasma on germination in different species.

**TABLE 1:** Cold plasma effects on germination in different species.

Plant species	Plasma treatment	Key results	References
*Arabidopsis thaliana*	Air plasma	Increased germination under osmotic and salinity stress; altered seed coat; improved stress tolerance	(Bafoil et al. 2019)
Soybean ( *Glycine max* )	Plasma at 120 W	Increase in germination; increase in vigor index; maximum effect at 80 W	(Ling et al. 2014)
Wheat ( *Triticum aestivum* )	Atmospheric cold plasma	Improved water uptake, drought tolerance, root and shoot growth, osmotic regulation	(Guo et al. 2017)
Barley ( *Hordeum vulgare* )	Direct plasma and plasma‐activated water	Increased soluble sugars, starch, protein; improved germination and seedling vigor	(Benabderrahim et al. 2024)
Fenugreek ( *Trigonella foenum‐graecum* )	Atmospheric cold plasma	Increased germination rate and seedling growth; enhanced physiological traits	(Fadhlalmawla et al. 2019)

### 3.2 Factors Affecting Plasma Performance

The type of gas used to generate plasma (e.g., argon, nitrogen, oxygen, helium, or air) plays a crucial role in determining the composition of reactive species. Increasing voltage or plasma power can intensify seed‐surface reactions and alter treatment outcomes. However, excessive settings may damage seeds and reduce viability, making careful optimization essential (Banu and Srinivasan 2026). The effectiveness of plasma treatment depends strongly on exposure time and intensity, exhibiting a dose‐dependent response. Short exposure durations generally stimulate germination and metabolic activity, whereas prolonged exposure or excessive intensity may lead to oxidative damage and cellular stress, ultimately inhibiting seed viability (Sera et al. 2010). Increasing voltage or plasma power enhances the generation of reactive oxygen species (ROS) and strengthens the electric field, which can further stimulate germination processes; however, excessive power levels may induce thermal or electrical damage, negatively affecting seed viability if critical thresholds are exceeded (Banu and Srinivasan 2026).

## 4 Cold Plasma in Inducing Resistance to Biotic Stresses

Seed contamination by fungi and bacteria can significantly reduce germination rates and seedling vigor, ultimately leading to lower crop yield and quality. In addition, contaminated seeds can act as carriers of plant pathogens, facilitating disease transmission and resulting in substantial agricultural losses (Amza 2018). Conventional chemical treatments (pesticides, antibiotics) are costly and environmentally harmful (Al‐Waili et al. 2012). Cold plasma, producing reactive oxygen and nitrogen species, can remove pathogens on seed surfaces while preserving germination capacity (Pańka et al. 2022). Figure 4 illustrates the multi‐step process of pathogen inactivation by cold plasma on seed surfaces.

**FIGURE 4:**
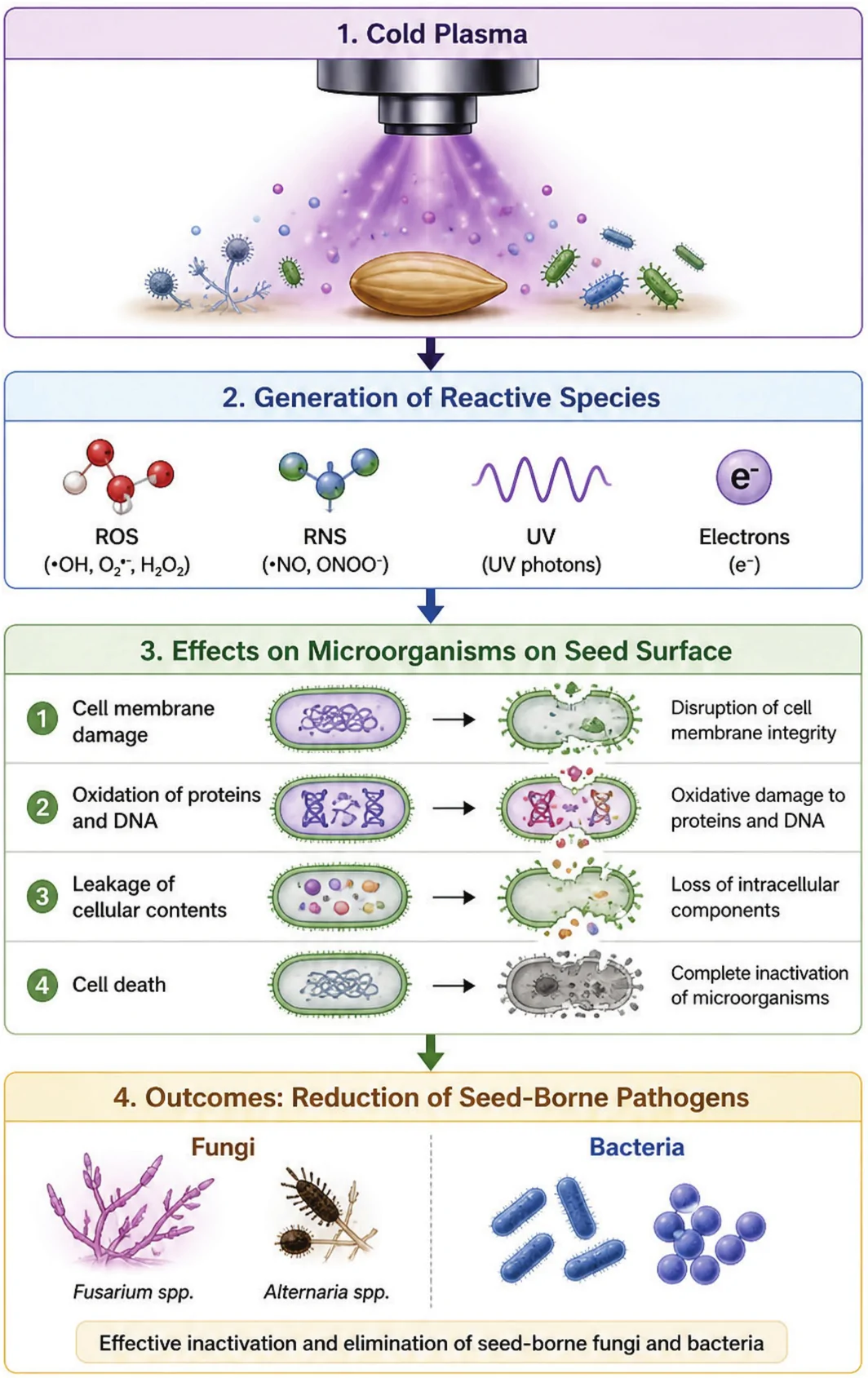
The multi‐step process of pathogen inactivation by cold plasma on seed surfaces.

Studies show cold plasma effectively disinfects seeds, fruits, vegetables, and cereals, providing a safe, eco‐friendly alternative to chemical disinfectants (Bora et al. 2022). The reactive radicals, ions, electrons, stable reaction products, active oxygen molecules, and UV radiation generated by cold plasma have antifungal effects (Jo et al. 2014). Hydroxyl radicals degrade microbial membranes and proteins, destroying cells (Bourke et al. 2017). Plasma efficacy also depends on the plasma device and operational parameters such as gas composition, flow rate, temperature, humidity, voltage, and frequency (Kolb et al. 2012).

Beyond their direct antimicrobial effects, cold plasma treatments may influence microbial interactions associated with seeds and the developing rhizosphere. However, a reduction in seed‐borne pathogens should not be interpreted as evidence of a predictable change in the wider microbial community. The effects of treatment on beneficial microorganisms, microbial diversity, and interactions among microbial groups remain insufficiently characterized. It is therefore unclear whether the observed antimicrobial effects lead to consistent changes in disease suppression or other ecological processes under field conditions. Addressing these questions will require studies that examine microbial community composition and function before and after treatment, preferably under realistic soil and growing conditions (Ivankov 2023).

Although plant‐pathogenic bacteria typically cause less economic damage than fungi, they can still compromise product quality. Plasma treatment has been shown to disrupt bacterial cell membranes and reduce viability in species such as *Staphylococcus aureus* and *Escherichia coli* , with Gram‐negative bacteria generally exhibiting greater sensitivity to plasma‐generated reactive species (Xu et al. 2018). Overall, cold plasma can eliminate fungi and bacteria on seed surfaces, enhancing crop yield, quality, and seed storage. In non‐disinfected winter wheat grain, cold plasma treatment reduced fungal colony counts by approximately 89.2% at an optimal exposure time of 10 s under the experimental conditions tested (Kordas et al. 2015).

Cold plasma also stimulates plant defense responses, activating antioxidant enzymes such as catalase (CAT), superoxide dismutase (SOD), peroxidase (POD), and increasing phenolic and flavonoid levels. In tomato, plasma treatment increased these enzymes 2–3 fold (Laroussi 2009). Plasma acts through mild UV, electric fields, and radical release, causing microbial cell leakage and death, like chemical‐free sterilization (Zhang et al. 2023). These reactive species can damage microbial cells and contribute to the activation of plant defense responses. A key advantage of cold plasma is complete disinfection without toxic residues, offering a sustainable alternative for organic agriculture (Niemira 2012).

## 5 Cold Plasma in Inducing Resistance to Abiotic Stresses

Plant stress encompasses any condition that limits a plant's ability to achieve its optimal growth, metabolic activity, and yield potential. Abiotic stress, in particular, results from non‐living environmental factors such as drought, salinity, temperature extremes, and heavy metal exposure. As sessile organisms, plants cannot escape these adverse conditions and must therefore adapt through coordinated morphological, cellular, and molecular responses to maintain survival and ecological fitness (Mahajan and Tuteja 2005). Adaptations include structural changes, physiological responses, protective compound production (e.g., antioxidants), phytohormone regulation, and activation of molecular defense systems (Souza et al. 2017). Humans have also sought to improve crop quality and protect plants using breeding, fertilizers, and genetic modification (Hansson and Joelsson 2013). Enhanced tolerance to abiotic stress may also influence plant fitness and survival under natural conditions, potentially affecting species distribution and adaptation to changing environments.

### 5.1 Salinity Stress

In saline conditions, sodium and chloride ions reduce osmotic potential, disrupt water uptake, and cause ionic toxicity. Cold plasma treatment increases seed coat wettability, facilitates water absorption, and stimulates defense pathways. Plasma has been shown to enhance germination rate, antioxidant enzyme activities (CAT, SOD), and reduce lipid peroxidation under salinity (Benabderrahim et al. 2024). Recent studies have demonstrated that plasma‐treated seeds exhibit lower sodium accumulation in shoot tissues and higher potassium/sodium ratios compared to untreated controls, indicating improved ionic homeostasis (Firouzkoohi et al. 2025).

### 5.2 Drought Stress

Drought limits growth during early plant establishment. Cold plasma‐treated seeds maintain water content, drought tolerance, and lower malondialdehyde (MDA) levels compared to controls by activating antioxidant systems and early defense pathways (Dufour et al. 2021). Plasma treatment has been shown to increase the accumulation of osmo‐protectants such as proline, glycine betaine, and soluble sugars, which help maintain cellular turgor under water deficit conditions. Studies report enhanced drought tolerance in various plants due to increased antioxidant enzyme activity, osmolyte accumulation, and reduced lipid peroxidation (Kumar et al. 2022). Plasma treatment improved germination and seedling growth in *Medicago sativa* and rapeseed under water stress (Feng et al. 2018).

### 5.3 Temperature Stress (Cold and Heat)

Temperature stress disrupts enzyme activity and uniform seedling growth. Cold plasma increases protective enzyme levels, stimulates protective compounds (e.g., proline), and enhances water uptake, improving tolerance to thermal stress. Plasma treatment has been shown to induce the expression of heat shock proteins (HSPs) and cold‐regulated (COR) genes, which play crucial roles in protecting cellular structures under temperature extremes. In maize and tomato, plasma increased germination rate and uniformity under suboptimal temperatures (Kamseu‐Mogo et al. 2025).

Temperature stress disrupts enzyme activity and uniform seedling growth. Cold plasma treatment can enhance protective enzyme activities and promote water uptake, thereby contributing to improved stress tolerance. Notably, Holubová et al. (2021) demonstrated that cold atmospheric plasma treatment of maize grains induced the expression of heat shock protein genes (HSP101 and HSP70) during the first 2 days after sowing (Holubová et al. 2021). In maize, Kamseu‐Mogo et al. (2025) reported that dry atmospheric plasma priming increased the germination rate from 65% to over 90% and reduced the median germination time by 37.5% at 15°C, indicating improved germination performance under suboptimal temperatures (Kamseu‐Mogo et al. 2025).

Under temperature stress, antioxidant enzymes help limit oxidative damage. Cold plasma treatment has been associated with enhanced antioxidant activity and improved seedling performance under suboptimal temperatures, although the magnitude of these effects depends on the plant species and treatment conditions (Song et al. 2020).

### 5.4 Heavy Metal Stress

Mahanta et al. (2022) reported that plasma‐activated water (PAW) improved germination and growth in soybean, and the presence of zinc oxide nanoparticles (ZnONPs) reduced lead (Pb) uptake by approximately fivefold in hydroponically grown seedlings (Mahanta et al. 2022).

## 6 Comparison of Cold Plasma With Other Seed Treatment Methods

Cold plasma, as a physical seed treatment, does not require chemical inputs, thereby reducing residue accumulation in both the environment and agricultural products (Benabderrahim et al. 2025). Its potential advantages include rapid treatment, reduced seed‐surface microbial contamination, and improved germination under suitable conditions (Banu and Srinivasan 2026; Sasikumar, Mangang, et al. 2025). However, practical adoption remains constrained by the need for specialized equipment, precise control of treatment parameters, and further validation under field conditions (Dufour et al. 2021). Table 2 summarizes the main advantages and disadvantages of cold plasma as a seed treatment method.

**TABLE 2:** Advantages and disadvantages of using cold plasma for seed treatment.

Category	Advantages	Disadvantages
Environmental	No need for chemicals, no toxic residues, suitable for organic farming (Desai et al. 2024)	Insufficient long‐term environmental impact studies on field scale (Dilip et al. 2026)
Efficiency and performance	Improved germination, enhanced seed vigor, increased activity of defense and metabolic enzymes (Benabderrahim et al. 2025)	Effectiveness depends on plant species and treatment conditions (species‐specific and parameter‐dependent) (Priatama et al. 2022)
Antimicrobial	Elimination of fungal and bacterial pathogens from seed surface without chemical fungicides (Lee et al. 2021)	Less effective in cases of deep or internal seed contamination (Ben Othman et al. 2024)
Time and speed	Fast process can be completed in a short time (Sasikumar, T, et al. 2025)	In some cases, repeated treatment may be required for full effectiveness (Waskow et al. 2021)
Technology and implementation	Scalable for industrial use, potential for combination with nanoparticles or other biological methods (Shelar et al. 2022)	Requires specialized equipment, high initial cost, precise standardization needed (Šimončicová et al. 2019)

## 7 Future Perspectives and Agricultural Applications

Cold plasma, as an innovative and sustainable agricultural technology, has significant potential to improve crop performance and reduce reliance on chemical inputs (Pańka et al. 2022). It offers an effective alternative to chemical pesticides and fertilizers, reducing production costs and increasing productivity, thereby contributing to sustainable agriculture.

Before cold plasma treatment can be widely adopted, several knowledge gaps need to be addressed. Further research is needed to determine how repeated treatments influence soil microbial communities and ecosystem processes under agricultural conditions. Treatment protocols also need to be standardized with respect to voltage, exposure duration, plasma source, and gas composition to improve reproducibility across plant species and cultivars. In addition, the persistence of treatment‐induced stress responses across successive generations remains an important question. Investigating whether these responses involve epigenetic regulation will require plant‐based experiments specifically designed to test the underlying mechanisms and distinguish persistent effects from environmental influences (Braný et al. 2021; Pal et al. 2025).

In parallel, integrating cold plasma with precision agriculture tools could enable more targeted and automated field applications. Scaling up plasma‐activated water (PAW) for irrigation and combining cold plasma with nanoparticles, phytohormones, and beneficial microbes may further enhance its agricultural effectiveness (Zou et al. 2025). Future studies should focus on the long‐term environmental impacts of this technology, standardization of physical parameters, and adaptation to real field conditions. Furthermore, integrating cold plasma with nanoparticles, phytohormones, bioactive compounds, and its application in smart agricultural systems opens new avenues for enhancing both the quality and quantity of agricultural products (Desai et al. 2024).

Several limitations of the available evidence should be considered when assessing the broader significance of cold plasma seed treatment. Most studies have been conducted under laboratory or controlled‐environment conditions, which may not fully reflect the variability of agricultural fields. Soil properties, changes in temperature and moisture, microbial interactions, and competition between neighboring plants can all influence plant responses under field conditions. Differences in plant species, seed characteristics, plasma systems, and exposure conditions also make comparisons between studies difficult. Moreover, improvements in germination, seedling growth, or antioxidant activity do not necessarily translate into greater survival, reproductive success, or final yield. Evidence concerning effects on plant community composition, soil microbial diversity and function, nutrient cycling, and other ecosystem processes remains limited. Addressing these gaps will require well‐designed field experiments and longer‐term studies that relate plant‐level responses to agricultural performance and environmental outcomes.

### 7.1 Barriers to Large‐Scale Field Adoption

Despite the promising laboratory results, the transition of cold plasma seed treatment from controlled experiments to large‐scale field adoption remains limited for several interrelated reasons. First, most plasma systems are designed for small batches, and achieving uniform, reproducible treatment across large seed volumes is difficult because plasma is primarily a surface phenomenon; seed loading, geometry, and shadowing can produce uneven exposure and inconsistent doses. Second, treatment outcomes depend strongly on reactor design and operational parameters, including gas composition, flow rate, humidity, voltage, frequency, exposure time, and distance. The absence of standardized dosimetry and reporting makes it difficult to compare studies, transfer protocols between species or cultivars, or define effective and safe treatment windows. Third, beneficial effects observed under controlled conditions often do not translate reliably to the field, where soil properties, temperature, moisture, microbial communities, and plant competition vary. Fourth, practical adoption is constrained by high initial equipment costs, energy consumption, maintenance requirements, limited throughput, and the need to integrate treatment into existing seed processing and storage systems. Fifth, regulatory frameworks, safety assessments, organic certification, and farmer acceptance remain uncertain, and the potential effects on beneficial seed and soil microorganisms are not well characterized. Consequently, large‐scale adoption remains limited not because the technology lacks potential, but because scalability, standardization, field performance, economic feasibility, and ecological safety have not yet been demonstrated sufficiently to justify widespread implementation.

### 7.2 Dose‐Dependence, Species‐Specificity, and Standardization

A major obstacle to industrial standardization is that cold plasma effects are strongly dose‐dependent and species‐specific, and often cultivar‐, seed‐lot‐, and environment‐dependent as well. Low or moderate exposure may stimulate germination and defense responses, whereas excessive exposure can cause oxidative damage and reduce viability, resulting in a narrow and context‐dependent treatment window. In addition, the optimal combination of plasma source, gas composition, voltage, frequency, exposure time, distance, humidity, and seed loading differs among plant species and even among cultivars. Because plasma is primarily a surface phenomenon, uniform treatment of large seed volumes is difficult, and scaling from laboratory reactors to industrial batch systems can alter the effective dose received by individual seeds. The lack of standardized dosimetry, reporting parameters, and biological response metrics further complicates comparisons across studies and prevents the direct transfer of protocols. Addressing these challenges will require standardized reporting guidelines, validated dosimetric measurements, response surface modeling, and predictive frameworks that can define effective and safe treatment windows for each crop and seed type. Machine learning and real‐time process monitoring could also help optimize parameters and maintain uniformity during scale‐up. Without such standardization, industrial adoption will remain limited, because reproducibility, quality control, and consistent efficacy are essential for commercial seed treatment.

A practical route towards field‐scale implementation should proceed in stages. Initial studies should identify a suitable treatment range for each crop and seed type, with sufficient information on plasma source, power, gas composition, exposure time, and seed loading to allow the procedure to be reproduced. The selected conditions should then be tested in larger seed batches to determine whether treatment remains uniform and whether any beneficial effects persist during storage. The next step is to evaluate treated seeds in replicated field trials across cultivars, locations, and growing seasons, using untreated seeds as controls and recording emergence, crop establishment, final yield, and relevant environmental variables. Before wider adoption, these trials should also assess energy consumption, processing capacity, equipment reliability, and treatment costs. This staged approach would help establish whether improvements observed under controlled conditions can be reproduced under agricultural conditions and whether the resulting benefits are sufficient to justify practical use.

The potential relationship between cold plasma treatment, stress memory, and evolutionary processes remains largely unresolved. Although treatment‐induced changes in plant physiology may persist beyond the initial response, it is not yet clear whether such effects are maintained across successive generations or influence heritable variation in plant populations. Establishing a role for epigenetic regulation would require direct evidence from appropriately controlled multigenerational experiments. Even if persistent effects were demonstrated, their consequences for adaptive plasticity, natural selection, and plant community dynamics would need to be evaluated separately. These questions provide directions for future research, but current evidence does not justify conclusions about evolutionary change or ecosystem‐level consequences resulting from cold plasma seed treatment (Ivankov 2023).

## 8 Conclusion

Cold plasma treatment has received growing attention as a method of improving seed performance. Studies have reported changes in seed germination, early seedling growth, pathogen resistance, and plant responses to environmental stresses. However, the results vary among plant species and are strongly influenced by treatment conditions and the environment in which plants are grown. Although these studies provide useful information about the responses of seeds and individual plants, much less is known about the effects of cold plasma on plant communities, soil microorganisms, biodiversity, and ecosystem functioning. Improved germination or greater tolerance to stress does not necessarily mean that the treatment will produce wider ecological benefits. It is also unclear whether changes induced by cold plasma persist beyond the initial treatment or are passed on to subsequent generations. Possible links with stress memory, transgenerational inheritance, and evolutionary change therefore require further investigation. Addressing these questions will require consistent treatment protocols, long‐term field studies, and experiments spanning several generations. Appropriate controls will also be needed to determine whether observed effects result from plasma treatment itself or from differences in environmental conditions and other sources of variation. Further studies should examine not only how cold plasma affects plant growth but also whether these effects influence interactions among plants, microorganisms, and other organisms in their surroundings. Such research would help clarify whether the benefits observed under controlled conditions translate into meaningful changes in agricultural systems and natural environments. For now, the broader ecological and evolutionary implications of cold plasma seed treatment remain uncertain and should be considered potential outcomes rather than established effects. Recognizing this limitation is important when assessing the practical value of the technology and its possible long‐term environmental consequences.

## Funding

The authors have nothing to report.

## Ethics Statement

There were no human subjects or animal experiments in our study.

## Conflicts of Interest

The authors declare no conflicts of interest.

## Data Availability

Data sharing not applicable to this article as no datasets were generated or analysed during the current study.
